# The telemedical imperative

**DOI:** 10.1111/bioe.12847

**Published:** 2021-02-15

**Authors:** Jordan A. Parsons

**Affiliations:** ^1^ Centre for Ethics in Medicine Bristol Medical School University of Bristol Bristol United Kingdom of Great Britain and Northern Ireland

**Keywords:** autonomy, health inequity, justice, remote healthcare, telemedicine

## Abstract

Technology presents a means of improving health outcomes for vast numbers of individuals. It has historically been deployed to streamline healthcare delivery and reach those who would previously have faced obstacles to accessing services. It has also enabled improved health education and management. Telemedicine can be employed in everything from primary care consultations to the monitoring of chronic diseases. Despite recommendation by the World Health Organization, countries have been slow to embrace such technology in the health sector. Nonetheless, it is expected to become more prevalent with increased digitization. Further, amidst the COVID‐19 pandemic, there was a rush to implement forms of telemedicine where possible to prevent patients breaking social distancing rules. In this paper, I present and defend what I term the ‘telemedical imperative’. The telemedical imperative represents a duty for healthcare systems to implement remote access to services where possible, thereby furthering the mission of equity in access to healthcare. It is intended as an addition to in‐person services rather than a replacement. After highlighting the benefits of telemedicine, I provide four criteria that must be met for the telemedical imperative to arise. The first three—safety, effectiveness, and acceptability—are consistent and essential. The fourth adapts to the service in question and requires that there be no other obstacles specific to that service that cannot reasonably be overcome. Finally, I address several potential objections to the telemedical imperative based on more general concerns around the implementation of telemedicine.

## 1 INTRODUCTION

Technology presents a means of improving health outcomes for vast numbers of individuals. Whether big data, artificial intelligence, or the internet of things, technology holds great promise in the health space.^1^ One such application is so‐called ‘telemedicine’. Telemedicine can be traced back over a century, with telephone consultations discussed in *The Lancet* as early as 1879.^2^ It has historically been deployed to streamline healthcare delivery and reach those who would previously have faced obstacles to accessing services. Indeed, in low‐ and middle‐income countries, telemedicine addresses health inequities by engaging those living in less affluent, rural communities.^3^ The broader classification of ‘telehealth’ also enables improved health education and management,^4^ although here I narrow my focus to telemedicine, meaning the use of technology in clinician–patient interactions for the purposes of healthcare delivery. Further, I focus only on services that are already provided in person, for reasons that will become apparent when I later highlight the importance of telemedicine becoming supplementary to in‐person provision and not a replacement for existing services.

Telemedicine can be employed in everything from primary care consultations^5^ to the monitoring of chronic diseases.^6^ Despite recommendation by the World Health Organization,^7^ countries have been slow to embrace such technology in the health sector. Nonetheless, it is expected to become more prevalent with increased digitization, with a 2019 report from Deloitte suggesting that ‘[w]ithin the next five years most people’s experience of accessing healthcare is likely to be digital‐first, primary care led’.^8^ Further, amidst the COVID‐19 pandemic, there has been (and remains) a rush to implement forms of telemedicine where possible.^9^ Ting and colleagues have even highlighted the potential for artificial intelligence to assist in the detection and diagnosis of COVID‐19.^10^ The swift introduction of telemedicine in response to the pandemic was to allow the overwhelming number of patients requiring medical attention that could feasibly be accessed remotely to do so, removing the need for such patients to break social distancing rules. The pandemic has therefore inadvertently and informally instigated an array of large‐scale trials of telemedicine globally—it will be important to assess these new services over time to determine the appropriateness of continuation when they are no longer essential to prevent the spread of the virus.

In this paper, I present and defend what I term the ‘telemedical imperative’ (TI). The TI represents a duty for healthcare systems to implement means of remote access to services where possible, thereby furthering the mission of equity in access to healthcare. Importantly, this is intended as an addition to in‐person services rather than a replacement. After highlighting the benefits of telemedicine, I provide four criteria that must be met for the TI to arise. The first three—safety, effectiveness, and acceptability—are consistent and essential. The fourth is adaptive to the service in question and requires that there be no other obstacles specific to that service that cannot reasonably be overcome. Finally, I address several potential objections to the TI based on more general concerns around the implementation of telemedicine.

## 2 BENEFITS OF TELEMEDICINE

### 2.1 Overcoming inequities

Many telemedical services focus on improving health outcomes for the most disadvantaged. In many cases this is in low‐ and middle‐income countries, where services are made available in communities that previously had no or limited means of accessing them. However, it is also the case in wealthier economies, where procedural barriers can prevent people from accessing healthcare. An early example in northern Norway was an initiative in the 1980s to allow citizens in rural communities that lacked specialist care services to access them remotely.^11^ Such systems would be especially useful in providing expertise for those with rare diseases (potentially across borders, although this would entail additional legal and regulatory considerations). Improving access remains necessary in almost all countries: whereas understandings of being geographically distant may differ across countries, in most there is a portion of the population for whom reaching the nearest health provider is neither quick nor easy. Even individuals living in close proximity to healthcare facilities may face obstacles in the form of childcare arrangements, time off work, or even anxiety in certain settings. Socioeconomic status is often a cause of these barriers, but even wealthier individuals and families may face them.

Telemedicine can contribute to overcoming these barriers and redress some of the socioeconomic imbalances that are closely related to health outcomes. Not only do so‐called postcode lotteries^12^ correlate with the urban–rural divide, it is also the case that the particular region in which a patient resides can present a challenge to accessing specialist care. Once implemented, telemedicine can ‘widen the capacity’ of clinicians in various specialties,^13^ thereby improving health outcomes in underserved areas. Not only are inequities addressed by removing these procedural barriers, but the financial savings that are sometimes made with the implementation of telemedicine can make possible an equitable redistribution of resources to further improve healthcare justice. Telemedicine has been found to be cost‐effective in various settings, including diabetic retinopathy screening in Singapore,^14^ orthopaedic consultations in Norway,^15^ and emergency medicine consultations in the United States.^16^ Of course, not all telemedical services will make clear financial sense, but where they do these savings might be redirected to, for example, establishing satellite clinics or providing patients who lack the means to access telemedicine with appropriate technology.

There is a concern that telemedicine can perpetuate and worsen the digital divide (which often correlates with socioeconomic status), which I will address shortly. For now, I simply wish to note that telemedicine has the *potential*, if correctly implemented, to overcome health inequities.

### 2.2 Efficiency

Waiting times for many healthcare services are long, and in some cases these delays can be harmful to patients. Not only might the health concern for which a patient is seeking medical attention worsen, but anxiety may increase because of delayed care.

Telemedicine has the potential to overcome these issues by improving efficiency. Telemedicine can improve efficiency not only from the provider’s side, but also from the patient’s side, as removing the need to travel to appointments lessens the time commitment for receiving care. This is especially beneficial in reaching those living remotely who would otherwise have to travel long distances. Even those who are not socioeconomically disadvantaged and could make these trips may well value the saved time.

Reduced waiting times can also improve safety.^17^ Patients could receive time‐sensitive treatments earlier and reduce the risk of complications. This applies to treatments that are time‐sensitive both in the medical and in the policy sense. Take, for example, gestational limits on access to abortion care—the limits imposed in many countries are not founded solely on medical evidence, even though the termination of pregnancy is safer and more effective earlier in pregnancy, and telemedicine makes it easier for women to access care before they exceed those limits.^18^ Reduced waiting times also contribute to disease prevention, as early signs may be noticed. In some cases, this could save lives.

Improved efficiency of service delivery has clear benefits across the board and demonstrates a need to at least *consider* the implementation of telemedicine where possible.

### 2.3 Enhancing autonomy

Telemedicine as additional to in‐person service delivery introduces a new option for patients. Rather than a binary choice between (in‐person) care or no care, patients would be presented with an additional option of telemedical care. The benefits of this to patients in terms of control over their daily lives may range from something as simple as being home for a delivery to avoiding the need to risk injury travelling to a GP if frail. These benefits may even be considered empowering.^19^ If a patient feels that they have more control over their care, this may result in more positive interactions with the healthcare system generally. The additional choice, therefore, is likely to be appreciated by many even if they do, either occasionally or always, opt for in‐person care.

Additional choices can be considered as enhancing a patient’s autonomy. Beauchamp and Childress write that a necessary condition of autonomy is that an agent be free of limitations that ‘prevent meaningful choice’.^20^ Assuming this procedural account, being presented with a yes/no choice in the context of medical care can be considered such a limitation as it is a false choice. Realistically, unless it is a minor ailment, few people would consider ‘no’ a genuine option. That is not to say that we should not consider consent to treatment valid purely because there was only one option. Rather, to be presented with additional options is to further enable meaningful choice. Having two options within the remit of ‘yes’ actually allows a choice, rather than a patient agreeing to the single option under the guise of a choice. It is necessary to remember here that the patient enters the clinician–patient relationship in a state of vulnerability because they require help, so it is important for the clinician to engage them in a decision‐making process;^21^ there is little process to deciding whether to accept the only option available.

Interestingly, Levy^22^ argues that *constrained* choices might in fact enhance autonomy, proposing mildly coercive modifications to informed consent that are designed to increase autonomy by guiding individuals towards their personal conception of the good. This reasoning might be taken to justify the provision of fewer choices. However, this is problematic when considering telemedicine as it would require an assumption that one option is preferable (meaning that it aligns with the individual preferences of all patients). Naturally, this is the case neither with in‐person nor with telemedical care. Whilst some patients will prefer in‐person services, others will view remote delivery as the best option. This does, of course, depend on how one interprets ‘constrained choices’. To offer four choices rather than six would be to constrain choices. Perhaps, then, offering in‐person and telemedical delivery of a service can still be considered a constraining of choices, as it is so relative to offering several in‐person options (for example, travel to a clinic that is far away or await the next visiting clinic) and several telemedical options (for example, videoconferencing consultation or chatbot) simultaneously. Understandably, to be overwhelmed with choice might undermine autonomy, as the individual may decide on a basis other than reason. Levy is then right in some circumstances. However, the addition of a telemedical option for healthcare services is not intended to introduce myriad options for patients but is a simple means of avoiding a false choice.

Whether or not the availability of a telemedical option actually *enhances* autonomy is a question of semantics: some might argue that it merely enables autonomy and that the absence of telemedicine limited autonomy. However one chooses to frame it, telemedicine at least enhances autonomy relative to the previous standard of care, as to constrain an individual’s choices is to undermine their autonomy at least to some extent.

Notably, this benefit does rely on telemedicine being an *additional* option rather than a replacement for in‐person services. In response to the COVID‐19 pandemic, for example, telemedicine has in some cases become a replacement and is not, therefore, enhancing autonomy. This, however, was a matter of necessity in unprecedented circumstances. These telemedical services may provide this particular benefit at a later date if they continue after the pandemic when there is a return to in‐person services. The risk of a decline in the provision of in‐person services—the telemedical primacy objection—is discussed shortly.

## 3 THE TELEMEDICAL IMPERATIVE

The TI is quad‐conditional (see Box 1) and provides something of a checklist for considering the introduction of telemedicine in the delivery of a given service. The first three conditions—safety, effectiveness, and acceptability—would generally be considered important in discussing any intervention whether telemedical or in person. They are necessary in respecting myriad ethical principles concerning the avoidance or minimizing of harm to patients. Should a telemedical service fail to satisfy these three, I argue that no such imperative exists. The fourth takes a different form depending on the service in question.

### 3.1 Safety

Of the three common criteria, safety is the most obvious. The importance of safety can be traced back to the Hippocratic Oath, whereby clinicians are duty bound not to harm their patients. This tradition has remained prominent in medical ethics and is now more commonly known as nonmaleficence.^23^ To suggest that an unsafe telemedical service be introduced is to firmly ignore this well‐established duty, making safety an important criterion. As Derse and Miller argue, telemedicine ‘should not be allowed to expand access at the expense of creating substandard practice’.^24^

A simple means of confirming that the safety criterion is satisfied is to consider the safety of the service provided by telemedicine relative to that in person; a relative standard is easier to apply than an objective one as it prevents the need to consider how safe is safe enough. After all, the TI is intended to apply to existing services, so a relative standard is feasible. Take, for example, an entirely verbal consultation with a GP. If the nature of the patient’s concern is not physically examinable, the GP provides advice based on the information the patient shares with them. It would be no safer for this conversation to take place in a surgery than on a video call, as the GP would still be able to ask any necessary questions and have the patient’s notes open. Therefore, such a consultation would easily satisfy the safety criterion.

There is a wealth of literature more specifically considering telepsychiatry. Freudenberg and Yellowlees provide an account of a case in which a patient’s quiet verbal indication of an intention to self‐harm was not picked up by the webcam he was using, so his doctor missed it.^25^ In this case, safety was a concern. Further, it highlights very clearly the importance of a good‐quality, stable connection. In the interests of safety, it would be appropriate for a clinician to bring a consultation to an end and either reschedule or insist on an in‐person consultation should the connection be poor.

An interesting point here is how the standard of care might apply from a legal perspective.^26^ Would it be appropriate for the standard of care to be lowered when a service is delivered remotely? Examples such as *Rudling* 27 suggest there are legal issues that would need addressing here. These issues vary across jurisdictions, so I will not deal with them extensively in this paper. Nonetheless, it is important to consider the question of whether care of a lower quality would be acceptable if it were to provide the benefits of telemedicine already outlined. Derse and Miller raise this question and suggest that quality care is a non‐waivable obligation.^28^ Given the importance of preventing undue harm to patients, I too suggest that the same standard ought to apply. Introducing telemedical services that are of lower quality would be to take one step forward and two steps back. However, the mantra of something is better than nothing might apply in certain, very limited, circumstances. Chaet and colleagues note the importance of considering what access a person might otherwise have to health care, citing examples such as a submarine crew and astronauts in space.^29^ In such extreme cases, it would be appropriate for telemedical services to be used even if the standard of care is lowered as a result. Certainly, the United States' National Aeronautics and Space Administration (NASA) has long been a leading force in the development of telemedicine.^30^ In ‘normal’ medical practice, however, this is not in keeping with the duty of clinicians to put patient welfare first.

### 3.2 Effectiveness

This second condition also partially concerns the avoidance of harm. If a telemedical service is ineffective—or less effective than that service delivered in person—the patient may be harmed. In that sense, the *physical* harm that may result from an ineffective telemedical service would also fall under the safety criterion. Effectiveness, however, goes further; a service may be safe but ineffective.

In many cases, the telemedical and in‐person delivery of a service will be comparably effective. A Cochrane Review found similar outcomes in conditions such as heart failure, as well as some evidence of improved quality of life with telemedicine.^31^ Another example is diabetes, which needs regular monitoring. Hjelm discusses diabetic patients taking their own blood glucose measurements whilst on a video call with a laboratory technician.^32^ This allows the results to be verified and removes the need for regular in‐person consultations. However, other conditions may not be as effectively managed remotely. Certain respiratory check‐ups, for example, may be unsuited to telemedicine. The control of asthma and chronic obstructive pulmonary disease requires the assessment of inhaler technique, which would be difficult on a video call, especially if the connection is less than perfect.

In some cases, the effective use of telemedicine may improve the effectiveness of other aspects of a patient’s care. Ossemane and colleagues discuss two SMS interventions in Mozambique that work to improve the retention of HIV+ patients in treatment.^33^ Interestingly, given that telemedicine is often implemented to reach those in rural areas, one of these interventions found success in urban patients but not rural patients.^34^

It must be noted, however, that just because a telemedical service is effective in general it is not necessarily effective in all situations. Clinical discretion is required in the telemedical provision of services that do, in principle, satisfy the criteria of the TI; it is for clinicians to decide on a case‐by‐case basis and, where they deem it necessary, insist on in‐person services for certain patients. As rightfully noted by Chaet and colleagues (on behalf of the American Medical Association’s Council on Ethical and Judicial Affairs), telemedicine is not appropriate in circumstances in which it prevents clinicians meeting established clinical standards.^35^ Policy can be responsible for wider decisions about the suitability of telemedicine for certain services, but individual decisions must ultimately come down to clinical judgement in the context of each patient; if a clinician feels that clinical standards will not be met using telemedicine with a patient, not only would it be acceptable to insist on in‐person delivery but it would be ethically required to prevent unnecessary harm. As Sabin and Skimming argue, it is important that services delivered by telemedicine maintain the same standards of ethics and professionalism as expected of in‐person services.^36^ Just as a patient cannot demand a treatment in person that their clinician does not consider appropriate, they cannot demand telemedical services either.

The effectiveness criterion is also about justice. If a service is ineffective when provided through telemedicine, that patient may require further medical attention in person—either the same service again, or further services due to a worsened condition. In such a situation, the use of telemedicine has not only resulted in unnecessary hassle for those involved, but also a greater use of resources, thereby risking an eventual increase in health inequities and a subsequent undermining of one of the key benefits of telemedicine. As such, the effectiveness criterion may also enable an economic case to be made where systems are well established, with IT systems of appropriate quality.

### 3.3 Acceptability

The third point to consider is how acceptable telemedical provision of a service is to patients. Again, the avoidance of harm partially guides this criterion, although this time it is a matter of potential psychological harm.

Acceptability is not only about tolerance of the physiological aspect of the service. In some circumstances, patient concerns about security may compromise acceptability. Some may worry that a telephone or video call may not be secure, or that a family member in the patient’s home will overhear personal information. Telemedical delivery of a service to such patients may cause unnecessary distress. This is most likely to be the case for especially sensitive issues or for patients with pre‐existing mental health conditions, but may be the case for others. However, it is necessary to note here that the reverse will also be true. Some patients may find GP or hospital waiting rooms distressing environments, thereby reaffirming the benefit of choice.

Concerns over acceptability may also arise in the case of informing relatives of a patient’s death and whether a video call, for example, is appropriate to try and console relatives. van Wynsberghe and Gastmans question whether consolation via computer would be done if the option of in person were available.^37^ The importance of communication to the clinician–patient (and, indeed, clinician–relative) relationship will be discussed shortly.

The extent of acceptability need not be overwhelming. A telemedical service that is preferred by a sizeable minority would satisfy the condition. Of course, if this were the case it would be important to make patients aware of the range of views, thereby providing appropriate information to allow them to decide if it is something that they might find acceptable. It is not reasonable to provide an exhaustive list of relative harms and information on acceptability, so a similar standard to in‐person care could be applied. For example, the test of materiality from the case of *Montgomery*,^38^ which requires that the clinician inform the patient of risks to which a reasonable person in the patient’s position would likely attach significance. It is because acceptability is a subjective question that the telemedical delivery of a service ought not to become the only—or even the default—option. Further, even if a patient finds the telemedical delivery of a service acceptable, that same patient may still prefer the in‐person equivalent.^39^

An important role of the acceptability criterion is to ensure that compassion is accounted for. Certain elements of care may be feasible remotely (and would be safe and effective) but still should rather clearly be delivered in person. An example is provided by Humbryd, drawn from an article in the New York Times.^40^ A patient, in a room with his family, was informed that he had incurable lung disease by a doctor he did not know via video link. Humbryd perfectly captures what is evident in this case—that the hospital was ‘ultimately choosing efficiency over compassion’.^41^

Cases like the one above demonstrate that telemedicine is not always appropriate. Just because it ‘works’, it does not mean that we should do it. It is reasonable to presume that most patients would not find receiving such information through telemedicine acceptable. Whilst there may be exceptions in some cases, compassion dictates that conversations informing patients that there is no more that can be done and/or that they have a very poor prognosis should take place in person. It is for this reason that safety and effectiveness alone do not make suitable criteria.

### 3.4 No service‐specific concerns

The fourth and final condition of the TI is rather more vague and its nature will differ between services. In essence, the criterion of no service‐specific concerns acts as a catch‐all to note that, in some circumstances, the fact that a telemedical service is safe, effective, and acceptable may not be reason enough to introduce it.

For example, in some circumstances the burden on carers/cohabitants may be significant enough to bring into question how ethical the use of telemedicine is. Bauer raises a concern that where complex medical equipment is placed in a patient’s home and monitored remotely, the home may become a ‘de facto ICU’ and place significant burden on the patient’s family.^42^ This would necessitate, argues Bauer, a family‐centred approach rather than the current standard patient‐centred approach to care decisions; it would be important to consider the impact on those living with the patient. Therefore, even if the placement of remotely monitored intensive care equipment in the home were to be safe, effective, and acceptable to the patient, the significant burden on others who are directly affected may cause it to fall short of this final criterion.

This example demonstrates the importance of service‐specific considerations as a final condition to the TI. Concerns about those close to the patient being burdened are not significant in many applications of telemedicine, such as online GP consultations and check‐ins with a specialist. The concern raised by Bauer is niche and seems only to apply to the uncommon circumstances of at‐home intensive care. It is for that reason that ‘burden to carers’ would not be an appropriate criterion in itself but can be encapsulated by this catch‐all when relevant.

By no means is this the only example. Myriad considerations may be dealt with as part of this fourth criterion. For example, legal concerns, resource constraints, and even the possibility of moral distress to clinicians. These are all important points but are not universally applicable; some telemedical services will be clearly within the requirements of legislation, cost‐saving, and pose no risk of moral distress.

One might contend that a catch‐all condition ignores the range of ethical questions raised by new telemedical services. However, its intention is quite the reverse. This fourth criterion serves to broaden the applicability of the TI. As with the de facto ICU example, many ethical concerns about specific telemedical services are somewhat unique to that service. This catch‐all acts to air these concerns without burdening consideration of all telemedical services with a range of unrelated ethical qualms. It can be adapted to meet the needs of the intervention in question, much like the process of specification that Beauchamp and Childress detail for the practical use of their four principles.^43^

## 4 SOME POTENTIAL OBJECTIONS

### 4.1 Telemedical primacy

The first objection I will consider is the possibility of a decline in in‐person services following the pursuit of the TI, and I will refer to it as the telemedical primacy objection.

One might worry that with a shift towards telemedicine, a gradual reduction in the availability of in‐person services may ensue. This response is understandable given how the digital age has caused a decline in other physical services—consider the impact of online shopping on the high street. Physical services are not always necessary and might only have been in person previously out of necessity (i.e., prior to widespread internet access). This, I suggest, is not the case in medicine.

Given this concern, it is important to stress that the TI does not entail the positioning of telemedical provision as the automatic mode of service delivery. Having demonstrated the myriad benefits of telemedicine, the question naturally arises as to whether it should be the default. If we allow repeat prescriptions to be obtained over the phone, should it only be on special request that a patient be allowed an in‐person consultation with their clinician to obtain one?

Duffy and Lee^44^ consider it a means of commitment to patient‐centred care to make in‐person services ‘Option B’. The ability of telemedicine to put a patient’s care at their fingertips, they argue, can improve the experience and streamline the whole system. However, this position contradicts one of the key benefits of telemedicine discussed earlier: its ability to enhance patient autonomy by offering choice. To make telemedicine the default not only increases the risk of it eventually becoming the only option but is likely to pressure patients into opting for it, thereby preventing meaningful choice. There are countless reasons why some patients would prefer to speak to a clinician face‐to‐face: elderly patients may not have internet at home; victims of domestic abuse may fear their abuser overhearing; and some patients may be anxious about the extent to which the means of communication are secure even after being reassured. To pressure such patients into video consultations and other telemedical services risks causing unnecessary anxiety. Instead, in‐person and telemedical options should be on an equal footing and both be offered to patients, allowing individual patients to decide which is preferable for them.

The TI seeks to improve healthcare—with particular attention to overcoming inequities—and to progress to a situation where only care that cannot be delivered through telemedicine is delivered in person does not further that goal. By introducing telemedicine as an *additional* service rather than the default, the risk of its becoming the only option is at least minimized. Certainly, this echoes the thoughts of hospice staff in a qualitative study about telehospice; whilst being positive overall about the use of telemedicine, there was a concern among several participants that it could interfere with in‐person services, resulting in the view that telemedicine should only be an additional option.^45^ As such, it is appropriate for clinicians to make patients aware of the option for telemedicine and to highlight its benefits. This must be done, however, as objectively as possible in order to avoid backtracking to paternalism, otherwise the benefit of enhanced autonomy is problematized.

### 4.2 Digital exclusion

A second objection, and one which naturally follows from the issue of telemedical primacy, is that of digital exclusion. There is a concern that some might be left behind owing to poor tech literacy or economic disadvantage if there is a push to implement telemedicine as much as possible.

This provides further justification for telemedicine being supplementary only. The removal of in‐person services in the push for telemedicine would perpetuate the digital divide that already exists in various areas of society. As a result, not only would the benefit of enhanced autonomy be lost, but the implementation of telemedicine would fail to further the goal of reducing inequity by simply shifting the access burden to a different population group.

Telemedicine being supplementary only, then, affords a benefit to those who can and choose to embrace it, whilst maintaining existing in‐person services prevents the care of those who neither can nor want to make use of telemedicine being worsened. Further, in developed countries at least, most people have internet access. For example, the Office for National Statistics reported that in 2019 93% of households in Great Britain had internet access.^46^ Depending on context, it may be appropriate for those who do not have internet access but would find it easier to receive care via telemedicine to be provided with appropriate equipment, much like programmes that provide disadvantaged students with laptops. This would be especially useful for patients with chronic conditions who have to attend regular consultations.

### 4.3 Damage to the clinician–patient relationship

One final objection is that telemedicine might damage the clinician–patient relationship. A lack of face‐to‐face interaction might prevent good communication,^47^ which some worry might negatively affect ‘trust, empathy, and overall patient outcomes’.^48^ Miller notes that non‐verbal signs are important to effective communication, and that without them the clinician–patient relationship may be damaged.^49^ Non‐verbal communication might include ‘voice quality and tone, eye contact, gaze, posture, laughter, facial expressions, body positioning, proximity, touch, activity (e.g. chart reviewing and computer usage) and other cues that modify the meaning of verbal utterances (e.g. hesitations)’.^50^ By not meeting a patient in person, a clinician may miss these signs. Not only might poorer communication itself damage the clinician–patient relationship, but it might result in, for example, a misdiagnosis that goes on to damage the relationship. Patients’ broader relationship with healthcare systems may also be damaged by the fragmentation of care among multiple providers.^51^

Of course, this does not *necessarily* have to be the case where services are delivered via telemedicine. Sabin and Skimming have highlighted several studies that (in the context of telepsychiatry) suggest that clinicians and patients have not found remote consultations to impede empathic connection.^52^ However, it must be acknowledged that some patients will feel that their communication with their clinician is hindered. Whereas some would jump at the opportunity to speak to their clinician from the comfort of their own home, others may suffer anxiety if forced to do so.^53^ As such, when providing care via telemedicine, special care must be taken to ‘convey appropriate social cues to reinforce the therapeutic relationship’.^54^

Additional training is certainly important to ensure this. In recognition of the increasing use of telemedicine, it is appropriate for medical schools to focus on the differences between bedside manner and telephone manner, and to ensure that clinicians are prepared to build rapport and instil necessary trust in their patients in the absence of face‐to‐face contact. Already‐qualified clinicians would also benefit from further education as telemedical services develop. There remains an obligation to provide competent care,^55^ and specific training can enable this.

Beyond highlighting the need for additional training, this final objection strengthens my argument that telemedical services ought only to be additional services and not replacements. Even with specific training to minimize, for example, the extent to which remote consultations impede good communication, some patients may still find it uncomfortable. To force such patients to attend remote consultations would itself damage the clinician–patient relationship, so it is important that in‐person options remain.

## 5 CONCLUSION

Given telemedicine’s potential for improving the healthcare of many, it is unsurprising that we are moving towards it. It is important, however, to consider the harms of the telemedicine rush. This is where the TI offers a simple assessment to establish the moral need to implement a particular service by telemedical means. Assuming the four criteria are met, telemedical provision of a service ought to be implemented.

Telemedicine is not a quick fix. I have highlighted ways in which it can overcome health inequities, but it cannot alone fix the underlying issues. However, telemedicine still has the potential to partially bridge the socioeconomic gap in health outcomes, and any money saved can—and should—be put towards closing the gap further through reaching those telemedicine does not.

Whilst my focus has been on medical practice, there is certainly a role for telemedicine in research, which might even increase participation. Global health, too, stands to benefit, although I have intentionally left this out of my discussion owing to the more specific issues that arise. For example, legal issues in cross‐border telemedicine.^56^ The TI in the global health context needs further discussion as a separate issue.

Implementing telemedicine is not without systemic obstacles, such as the difficulty of fitting it into routine practice and resistance to change.^57^ Cross‐border telemedicine may also prove to be an intranational obstacle in countries such as the United States, where certain medical regulation is at the state level.^58^ Undoubtedly, the initial introduction of a telemedical service will encounter minor problems. However, this is not reason to deny the TI; it does not problematize the fourth criterion. We must pursue the expansion of telemedicine both to enhance patient autonomy and to improve health outcomes by redressing longstanding inequities.

## CONFLICT OF INTEREST

The author declares no conflict of interest.

**Conditions of the telemedical imperative.**

- *Safety:* In line with the duty to ‘do no harm’, telemedical services must present no significant risk of harm relative to in‐person delivery of the same service.
- *Effectiveness:* The effectiveness of a service provided by telemedicine must be similar to that of the same service provided in person.
- *Acceptability:* Patients must consider telemedical delivery of a service acceptable, although it need not be a majority.
- *No service‐specific concerns:* In addition to the three general criteria, there must be no significant concerns that are specific to the service in question that cannot satisfactorily be overcome.

## Footnotes

^1^
Ting, D. S. W., Carin, L., Dzau, V., & Wong, T. Y. (2020). Digital technology and COVID‐19. *Nature Medicine*, *26*, 459–461.10.1038/s41591-020-0824-5PMC710048932284618

^2^
_____. (1879). Notes, short comments, and answers to correspondents. *Lancet*, *114*(2935), P819–822.

^3^
Tyagi, N., Goel, S. A., & Alexander, M. (2019). Improving quality of life after spinal cord injury in India with telehealth. *Spinal Cord Series and Cases*, *5*, 70.10.1038/s41394-019-0212-xPMC678631131632728

^4^
Conde, J. G., De, S., Hall, R. W., Johansen, E., Meglan, D., & Peng, G. C. Y. (2010). Telehealth innovations in health education and training. *Telemedicine and e‐Health*, *16*(1), 103–106.10.1089/tmj.2009.0152PMC293734620155874

^5^
Raven, M., Butler, C., & Bywood, P. (2013). Video‐based telehealth in Australian primary health care: Current use and future potential. *Australian Journal of Primary Healthcare*, *19*(4), 283–286.10.1071/PY1303224134865

^6^
Rohatgi, R., Ross, M. J., & Majoni, S. W. (2017). Telenephrology: Current perspectives and future directions. *Kidney International*, *92*(6), 1328–1333.10.1016/j.kint.2017.06.03228893419

^7^
World Health Organization. (2010). *Telemedicine: Opportunities and developments in member states*. Geneva: WHO Press.

^8^
Deloitte Centre for Health Solutions. (2019). *Closing the digital gap: Shaping the future of UK healthcare*. Retrieved from https://www2.deloitte.com/content/dam/Deloitte/uk/Documents/life‐sciences‐health‐care/deloitte‐uk‐life‐sciences‐health‐care‐closing‐the‐digital‐gap.pdf

^9^
British Medical Association. (2020). *COVID‐19: Video consultations and homeworking*. Retrieved from https://www.bma.org.uk/advice‐and‐support/covid‐19/adapting‐to‐covid/covid‐19‐video‐consultations‐and‐homeworking; Wosik, J., Fudim, M., Cameron, B., Gellad, Z. F., Cho, A., Phinney, D., … Tcheng, J. (2020). Telehealth transformation: COVID‐19 and the rise of virtual care. *Journal of the American Medical Informatics Association*, *27*(6), 957–962.10.1093/jamia/ocaa067PMC718814732311034

^10^
Ting et al., op. cit. note 1.

^11^
Elford, D. R. (1997). Telemedicine in northern Norway. *Journal of Telemedicine and Telecare*, *3*, 1–22.10.1258/13576339719301399139756

^12^
Stanberry, B. (2006). Legal and ethical aspects of telemedicine. *Journal of Telemedicine and Telecare*, *12*, 166–175.10.1258/13576330677748882516774696

^13^
Dickens, B., & Cook, R. (2006). Legal and ethical issues in telemedicine. *International Journal of Gynaecology and Obstetrics*, *94*, 73–78, p. 77.10.1016/j.ijgo.2006.04.02316777109

^14^
Nguyen, H. V., Tan, G. S., Tapp, R. J., Mital, S., Ting, D. S., Wong, H. T., … Lamoureaux, E. L. (2016). Cost‐effectiveness of a national telemedicine diabetic retinopathy screening program in Singapore. *Opthalmology*, *123*(12), 2571–2580.10.1016/j.ophtha.2016.08.02127726962

^15^
Buvik, A., Bergmo, T. S., Bugge, E., Smaabrekke, A., Wilsgaard, T., & Olsen, J.A. (2019). Cost‐effectiveness of telemedicine in remote orthopedic consultations: Randomized controlled trial. *Journal of Medical Internet Research*, *21*(2), e11330.10.2196/11330PMC639957230777845

^16^
Nord, G., Rising, K. L., Band, R. A., Carr, B. G., & Hollander, J. E. (2019). On‐demand synchronous audio video telemedicine visits are cost effective. *American Journal of Emergency Medicine*, *37*(5), 890–894.10.1016/j.ajem.2018.08.01730100333

^17^
Hjelm, N. M. (2005). Benefits and drawbacks of telemedicine. *Journal of Telemedicine and Telecare*, *11*, 60–70.10.1258/135763305349988615829049

^18^
Romanis, E. C., Parsons, J. A., & Hodson, N. (2020). COVID‐19 and reproductive justice in Great Britain and the United States: Ensuring access to abortion care during a global pandemic. *Journal of Law and the Biosciences*, *7*(1), lsaa027.10.1093/jlb/lsaa027PMC731385932728469

^19^
Kaplan, B., & Litewka, S. (2008). Ethical challenges of telemedicine and telehealth. *Cambridge Quarterly in Healthcare Ethics*, *17*, 401–416.10.1017/S096318010808053518724880

^20^
Beauchamp, T. L., & Childress, J. F. (2013) *Principles of biomedical ethics* (7th ed). New York, NY: Oxford University Press, p. 101.

^21^
van Wynsberghe A., & Gastmans C. (2008) Telesurgery: An ethical appraisal. *Journal of Medical Ethics*, *34*, e22.10.1136/jme.2007.02395218827095

^22^
Levy, N. (2014). Forced to be free? Increasing patient autonomy by constraining it. *Journal of Medical Ethics*, *40*, 293–300.10.1136/medethics-2011-100207PMC399528722318413

^23^
Beauchamp & Childress, op. cit. note 20.

^24^
Derse, A. R., & Miller, T. E. (2008). Net effect: Professional and ethical challenges of medicine online. *Cambridge Quarterly of Healthcare Ethics*, *17*(4), 453–464.10.1017/S096318010808057218724884

^25^
Freudenberg, N., & Yellowlees, P. M. (2014). Telepsychiatry as part of a comprehensive care plan. *American Medical Association Journal of Ethics*, *16*(12), 964–968.10.1001/virtualmentor.2014.16.12.ecas2-141225493364

^26^
Stanberry, op. cit. note 12.

^27^

*R v Rudling* [2016] EWCA Crim 741.

^28^
Derse & Miller, op. cit. note 24, p. 458.

^29^
Chaet, D., Clearfield, R., Sabin, J. E., & Skimming, K. (2017). Ethical practice in telehealth and telemedicine. *Journal of General Internal Medicine*, *32*(10), 1136–1140, p. 1137.10.1007/s11606-017-4082-2PMC560275628653233

^30^
Nicogossian, A. E., Pober, D. F., & Roy, S. A. (2001) Evolution of telemedicine in the space program and Earth applications. *Telemedicine Journal and e‐Health*, *7*(1), 1–15.10.1089/15305620130009381311321704

^31^
Flodgren, G., Rachas, A., Farmer, A. J., & Inzitari, M. (2015). Interactive telemedicine: Effects on professional practice and health care outcomes. *Cochrane Database of Systematic Reviews*, *9*, CD002098.10.1002/14651858.CD002098.pub2PMC647373126343551

^32^
Hjelm, op. cit. note 17.

^33^
Ossemane, E. B., Moon, T. D., Were, M. C., & Heitman, E. (2017). Ethical issues in the use of SMS messaging in HIV care and treatment in low‐ and middle‐income countries: Case examples from Mozambique. *Journal of the American Medical Informatics Association*, *25*(4), 423–427.10.1093/jamia/ocx123PMC588580029088384

^34^
Joseph Davey, D., Nhavoto, J. A., Augusto, O., Ponce, W., Traca, D., Nguimfack, A., & Palha de Sousa, C. (2016). SMSaúde: Evaluating mobile phone text reminders to improve retention in HIV care for patients on antiretroviral therapy in Mozambique. *Journal of Acquired Immune Deficiency Syndrome*, *73*(2), e23–e30.10.1097/QAI.0000000000001115PMC576727827632147

^35^
Chaet et al., op. cit. note 29, p. 1137.

^36^
Sabin J. E., & Skimming, K. (2015). A framework of ethics for telepsychiatry practice. *International Review of Psychiatry*, *27*(6), 490–495, p. 494.10.3109/09540261.2015.109403426493214

^37^
van Wynsberghe, A., & Gasmans, C. (2009). Telepsychiatry and the meaning of in‐person contact: A preliminary ethics appraisal. *Medicine, Health Care and Philosophy*, *12*, 469–476, p. 473.10.1007/s11019-009-9214-y19629748

^38^

*Montgomery v Lanarkshire Health Board* [2015] UKSC 11.

^39^
Collins, K., Nicolson, P., & Bowns, I. (2000). Patient satisfaction in telemedicine. *Health Informatics Journal*, *6*, 81–85; Chaet et al., op. cit. note 29, p. 1137.

^40^
Humbyrd, C. S. (2019). Virtue ethics in a value‐driven world: Ethical telemedicine. *Clinical Orthopaedics and Related Research*, *477*(12), 2639–2641, p. 2639.10.1097/CORR.0000000000000908PMC690728731764325

^41^
Ibid.

^42^
Bauer, K. A. (2001). Home‐based telemedicine: A survey of ethical issues. *Cambridge Quarterly in Healthcare Ethics*, *10*, 137–146, p. 141.10.1017/s096318010100204311302090

^43^
Beauchamp & Childress, op. cit. note 20, p. 17.

^44^
Duffy, S., & Lee, T. H. (2018). In‐person health care as option B. *New England Journal of Medicine*, *378*, 104–106.10.1056/NEJMp171073529320653

^45^
Demiris, G., Parker Oliver, D. R., Fleming, D. A., & Edison, K. (2004). Hospice staff attitudes towards telehospice. *American Journal of Hospice & Palliative Medicine*, *21*(5), 343–347, p. 346.10.1177/10499091040210050715510570

^46^
Office for National Statistics. (2019). *Internet access – households and individuals, Great Britain: 2019*. Retrieved from https://www.ons.gov.uk/peoplepopulationandcommunity/householdcharacteristics/homeinternetandsocialmediausage/bulletins/internetaccesshouseholdsandindividuals/2019

^47^
Silverman, R. D. (2003) Current legal and ethical concerns in telemedicine and e‐medicine. *Journal of Telemedicine and Telecare*, *9*(s1), 67–69.10.1258/13576330332219640212952729

^48^
Bauer, op. cit. note 42, p. 142.

^49^
Miller E. A. (2003). The technical and interpersonal aspects of telemedicine: Effects on doctor–patient communication. *Journal of Telemedicine and Telecare*, *9*(1), 1–7.10.1258/13576330332115961112641885

^50^
Ibid: 3–4.

^51^
Dorsey, E. R., & Topol, E. J. (2016). State of telehealth. *New England Journal of Medicine*, *375*, 154–161, p. 157.10.1056/NEJMra160170527410924

^52^
Sabin & Skimming, op. cit. note 36, p. 491.

^53^
Clarke, P. A., Capuzzi, K., & Harrison, J. (2010). Telemedicine: Medical, legal and ethical perspectives. *Medical Science Monitor*, *16*(12), RA261–RA272.21119593

^54^
Terrasse, M., Gorin, M., & Sisti, D. (2019). Social media, e‐health, and medical ethics. *Hastings Center Report*, *49*(1), 24–33, p. 26.10.1002/hast.97530790306

^55^
Chaet et al., op. cit. note 29, p. 1138.

^56^
Dickens & Cook, op. cit. note 13.

^57^
de Bont, A., & Bal, R. (2008). Telemedicine in interdisciplinary work practices: On an IT system that met the criteria for success set out by its sponsors, yet failed to become part of every‐day clinical routines. *BMC Medical Informatics and Decision Making*, *8*, 4710.1186/1472-6947-8-47PMC261574918954428

^58^
Stanberry, op. cit. note 12.

